# Nonlinear Free and Forced Vibrations of a Hyperelastic Micro/Nanobeam Considering Strain Stiffening Effect

**DOI:** 10.3390/nano11113066

**Published:** 2021-11-14

**Authors:** Amin Alibakhshi, Shahriar Dastjerdi, Mohammad Malikan, Victor A. Eremeyev

**Affiliations:** 1 Department of Mechanical Engineering, Science and Research Branch, Islamic Azad University, Tehran 1477893855, Iran; alibakhshiamin@yahoo.com; 2 Civil Engineering Department, Division of Mechanics, Akdeniz University, Antalya 07058, Turkey; dastjerdi_shahriar@yahoo.com; 3 Department of Mechanics of Materials and Structures, Faculty of Civil and Environmental Engineering, Gdansk University of Technology, 80-233 Gdansk, Poland; victor.eremeev@pg.edu.pl; 4 Department of Civil and Environmental Engineering and Architecture (DICAAR), Università degli Studi di Cagliari, Via Marengo 2, 09123 Cagliari, Italy

**Keywords:** hyperelastic micro/nanobeam, extended modified couple stress theory, strain-stiffening effect, nonlinear frequency response

## Abstract

In recent years, the static and dynamic response of micro/nanobeams made of hyperelasticity materials received great attention. In the majority of studies in this area, the strain-stiffing effect that plays a major role in many hyperelastic materials has not been investigated deeply. Moreover, the influence of the size effect and large rotation for such a beam that is important for the large deformation was not addressed. This paper attempts to explore the free and forced vibrations of a micro/nanobeam made of a hyperelastic material incorporating strain-stiffening, size effect, and moderate rotation. The beam is modelled based on the Euler–Bernoulli beam theory, and strains are obtained via an extended von Kármán theory. Boundary conditions and governing equations are derived by way of Hamilton’s principle. The multiple scales method is applied to obtain the frequency response equation, and Hamilton’s technique is utilized to obtain the free undamped nonlinear frequency. The influence of important system parameters such as the stiffening parameter, damping coefficient, length of the beam, length-scale parameter, and forcing amplitude on the frequency response, force response, and nonlinear frequency is analyzed. Results show that the hyperelastic microbeam shows a nonlinear hardening behavior, which this type of nonlinearity gets stronger by increasing the strain-stiffening effect. Conversely, as the strain-stiffening effect is decreased, the nonlinear frequency is decreased accordingly. The evidence from this study suggests that incorporating strain-stiffening in hyperelastic beams could improve their vibrational performance. The model proposed in this paper is mathematically simple and can be utilized for other kinds of micro/nanobeams with different boundary conditions.

## 1. Introduction

For many decades, vibration analysis of mechanical structures was a major topic among scientists [1,2,3,4,5,6,7,8,9,10]. Over recent decades, a surge of interest in studying hyperelastic materials was shown. The main characteristic of hyperelastic materials is that their strain-stress diagram is nonlinear and may undergo large deformations [11,12,13]. Hyperelastic materials play a vital role in soft systems and structures, e.g., soft robotics [14], human organs [15,16], soft actuators [17,18], soft sensors [19,20], and soft energy harvesters [19,20,21,22]. Data from previous studies show that various mechanical structures such as beams, plates, membranes, and shells were made of hyperelastic materials [23,24,25,26,27,28,29,30,31,32,33,34,35]. It was reported that hyperelastic beams are an appropriate candidate to fabricate systems with high performance. For this reason, this paper focuses on beams made of hyperelastic materials. In light of the applications and properties of hyperelastic beams, it is becoming extremely difficult to ignore their investigation in different situations.

There are numerous published works that investigate dependent and time-independent responses of hyperelastic beams. For example, the nonlinear postbuckling of a hyperelastic beam-like structure was investigated by Lubbers et al. [36]. They employed the neo-Hookean hyperelastic model in conjunction with empirical tests and the finite element technique in their study. Wang and coworkers [37] studied nonlinear vibration of hyperelastic beams utilizing time history diagrams and frequency responses, who employed a compressible neo-Hookean constitutive law. He and coworkers [38] developed the Euler–Bernoulli beam model in a new finite strain framework to model a neo-Hookean hyperelastic beam. Xu and Liu [39] proposed an improved method to dynamically explore the response of a beam-like hyperelastic structure, where a Yeoh model was utilized to capture the material nonlinearity. Nonlinear dynamic characteristics of a soft hyperelastic beam were investigated by Wang et al. [40], employing a compressible neo-Hookean model and variational approach. Wang and Zhu [41] studied the nonlinear oscillation of a harmonically excited hyperelastic beam. They utilized the frequency-amplitude response, time histories, and a compressible neo-Hookean model in their investigation. The finite bending of a beam made of hyperelastic materials was analyzed by Bacciocchi and Tarantino [42]. They utilized a compressible Mooney-Rivlin hyperelastic material model to physical nonlinearity of the beam. Dadgar–Rad and Sahraee [43], by considering the incompressibility condition, investigated the large deformation response of a beam made of hyperelastic materials, where a neo-Hookean model was employed as the hyperelastic constitutive model. Bacciocchi and Tarantino [44] conducted a finite anticlastic bending analysis of hyperelastic beams using two hyperelastic models, namely Mooney–Rivlin and Saint Venant–Kirchhoff. Lanzoni and coworker [45] studied the nonuniform bending of a beam made of the hyperelastic beam, taking the Mooney–Rivlin into account. The large deformation response of hyperelastic beams was explored by Dadgar–Rad and Firouzi [46]. They incorporated Fung’s quasilinear viscoelasticity theory and Mooney-Rivlin model.

Results from earlier studies demonstrate that few researchers addressed the modelling of hyperelastic beams with the strain-stiffening effect. Furthermore, previous studies have notably investigated a beam-like hyperelastic structure on a large scale and have not considered the hyperelastic beams in micro/nanoscales. However, fabrication of such beams in smaller scales was feasible, and hence analyzing hyperelastic micro/nanobeam and proposing more sophisticated theories should be developed for such structures. A challenging problem that arises in this domain is accurate modelling for hyperelasticity in micro/nanoscales. More specifically, in nanoscale, it is necessary to capture the size effect. Because hyperelastic materials may undergo large deformation and large rotation, these conditions should be considered on micro/nanoscale. One of the problems that it investigates in hyperelasticity is the strain-stiffening effect. This effect may improve or limit the performance of hyperelastic micro/nanobeams. Therefore, incorporating strain-stiffening with simple mathematical modelling in micro/nanoscale is essential. Specifically, to our knowledge, no study has considered large deformation, strain-stiffening, and moderate rotation for hyperelastic micro/nanobeams.

This paper aims to propose a sophisticated model for a micro/nanobeam made of hyperelastic materials that incorporate the small-scale and strain-stiffening effects of nonlinear elasticity. The nonlinear equations of motion are derived via Hamilton’s principle and an extended von-Kármán theory. The frequency-amplitude plot and nonlinear resonance plot are presented by considering different system parameters. The results are discussed in detail, and influential parameters on free and forced vibrations of the hyperelastic micro/nanobeam are identified.

## 2. Governing Equations

**Figure 1:**
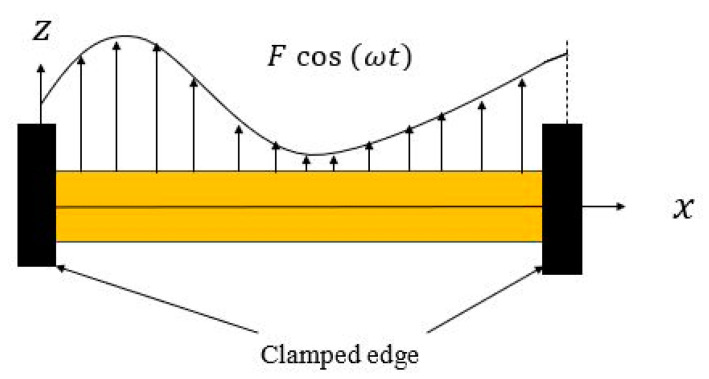
Schematic representation of a clamped-clamped hyperelastic beam.

The schematic view of the hyperelastic micro/nanobeam is illustrated in Figure 1, where the length, width, and height of the beam are denoted by L, b, and d, respectively. A clamped-clamped boundary condition is assumed to the beam, and a harmonic transverse mechanical load is applied to it. It is considered that the length of the beam is much greater than the depth. In addition, the shear deformation and rotary inertia are neglected. Thus, we use the Euler–Bernoulli (E-B) beam theory to define the displacement field.

The displacement field for the beam is established according to the Euler–Bernoulli beam equation, namely,

(1) $$u_{x}=-z\frac{\partial w\left(x,t\right)}{\partial x}\;u_{y}=0\;u_{z}=w\left(x,t\right)$$

where w(x,t) stands for the transverse displacement of any point on the neutral axis. The strain-displacement relations originated for the Euler–Bernoulli beam theory are modelled based on an extended von Kármán equation, in which large deformation, moderate rotation, and transverse strain are included, namely [47,48]

(2) $$\varepsilon_{1}=\frac{1}{2}{\left(\frac{\partial w}{\partial x}\right)}^{2}-z\frac{\partial^{2}w}{\partial x^{2}}\;\varepsilon_{3}=\frac{1}{2}\;{\left(\frac{\partial w}{\partial x}\right)}^{2}$$

Other components of the extended von Kármán equation are equal to zero.

The strain energy of the hyperelastic micro/nanobeam is decomposed into two parts, i.e., the potential due to the hyperelasticity and the potential due to small-scale effects.

For hyperelastic materials, a strain energy function is used to obtain the strain energy of the system. Numerous hyperelastic strain energy functions can capture the strain stiffening, for instance, the standard Gent, the Arruda–Boyce, and modified versions of the Standard Gent model [49,50,51]. In this work, for simplicity, a standard Gent model is considered, in which the strain-stiffening effect is incorporated, namely [52,53]

(3) $$\Psi_{1}=\frac{\mu }{2}\left[\left(I_{1}-3\right)+\frac{1}{2\;J_{m}}{\left(I_{1}-3\right)}^{2}+\ldots +\frac{1}{\left(n+1\right)J_{m}^{n}}{\left(I_{1}-3\right)}^{n+1}\right]$$

where μ is the shear modulus; $I_{1}$ denotes the first invariant of the deformation tensor; $J_{m}$ is a dimensionless parameter that is called the stiffening parameter.

For simplicity, the second-order expansion of the standard Gent model is utilized, such that

(4) $$\Psi_{1}=\frac{\mu }{2}\left[\left(I_{1}-3\right)+\frac{1}{2\;J_{m}}{\left(I_{1}-3\right)}^{2}\right]$$

The first principal invariant of the right Cauchy–Green deformation tensor in terms of the extended von Kármán strains is formulated as [54]

(5) $$I_{1}=2\left(\varepsilon_{1}+\varepsilon_{2}+\varepsilon_{3}\right)+3$$

Substituting Equation (2) into Equation (5), the first principal invariant is reformulated as

(6) $$I_{1}=2\left[{\left(\frac{\partial w}{\partial x}\right)}^{2}-z\frac{\partial^{2}w}{\partial x^{2}}\right]+3$$

Substituting Equation (6) into Equation (4), the Gent strain energy function as a function of transverse displacement is obtained below

(7) $$\Psi_{1}=\int_{0}^{L}\left[\mu A{\left(\frac{\partial w}{\partial x}\right)}^{2}+\frac{\mu A}{J_{m}}{\left(\frac{\partial w}{\partial x}\right)}^{4}+\frac{\mu }{J_{m}}I{\left(\frac{\partial^{2}w}{\partial x^{2}}\right)}^{2}\right]dx$$

It is mentioned that Equation (7) was obtained by considering the following relations

(8) $$I=\int_{A}z^{2}\;dydz=b\frac{d^{3}}{12}\;A=\int_{A}\;dydz=bd\;0=\int_{A}\;z\;dydz$$

In Equation (8), A is the cross-section area, and I represents the second moment of the cross-section.

The potential of the small-scale effect is considered through the use of an extended modified couple stress theory, such that [47]

(9) $$\Psi_{2}=\frac{1}{2}\left(2\mu A\ell^{2}\right)\int_{0}^{L}{\left(\frac{\partial^{2}w}{\partial x^{2}}\right)}^{2}dx$$

where ℓ is a length-scale parameter.

Comparing Equation (9) with previous studies, for the moderate rotation, a coefficient 2 appears in the equation in comparison to the small rotation [55].

Finally, the total strain energy of the hyperelastic micro/nanobeam is written as

(10) $$U_{s}=\Psi_{1}+\Psi_{2}$$

The moving beam generates the kinetic energy in the system, which is formulated as

(11) $$U_{k}=\frac{1}{2}\rho A\int_{0}^{L}{\left(\frac{\partial w}{\partial t}\right)}^{2}dt$$

where ρ stands for the mass-density of the hyperelastic beam.

The transverse applied periodic loading does the work of the following form

(12) $$W_{F}=\int_{0}^{L}F\cos\left(\omega t\right)w\;dx$$

in which F is the amplitude and ω indicates the excitation frequency.

The work generated from the viscous damping is expressed as

(13) $$W_{D}=-c_{D}\;\int_{0}^{L}\frac{\partial w}{\partial t}w\;dx$$

where $c_{D}$ is the viscous damping coefficient.

To derive boundary conditions and governing equation, Hamilton’s principle is utilized, namely

(14) $$\delta \;\int_{t_{1}}^{t_{2}}[U_{k}-U_{S}]dt+\delta \;\int_{t_{1}}^{t_{2}}\left[\delta W_{F}+\delta W_{D}\right]dt=0$$

Substituting Equations (10)–(13) into Equation (14), we obtain the following equations

(15) $$\rho A\frac{\partial^{2}w}{\partial t^{2}}+C_{D}\frac{\partial w}{\partial t}+\frac{2\mu I}{J_{m}}\frac{\partial^{4}w}{\partial x^{4}}+2\mu A\ell^{2}\frac{\partial^{4}w}{\partial x^{4}}-2\mu A\frac{\partial^{2}w}{\partial x^{2}}-\frac{12\mu A}{J_{m}}{\left(\frac{\partial w}{\partial x}\right)}^{2}\frac{\partial^{2}w}{\partial x^{2}}=F\cos\left(\omega t\right)$$

and boundary conditions for the double-clamped micro/nanobeam

(16) $$w\left(0\right)=0,\;w\left(L\right)=0,\;\frac{dw\left(0\right)}{dx}=0,\;\frac{dw\left(L\right)}{dx}=0$$

The above equations are made dimensionless to simplify and generalize the vibration analysis of the micro/nanobeam. The following nondimensional quantities are introduced, such that

(17) $$\hat{x}=\frac{x}{L},\;\hat{w}=\frac{w}{L},\;\hat{t}=t\sqrt{\frac{\mu I}{\rho AL^{4}}},\;\hat{c}=\frac{cL^{4}}{\mu I}\sqrt{\frac{\mu I}{\rho AL^{4}}},\;\Omega =\omega \sqrt{\frac{\rho AL^{4}}{\mu I}}\;\eta_{1}=\frac{2\mu A\ell^{2}}{\mu I},\;\eta_{2}=-\frac{2\mu AL^{2}}{\mu I},\;\beta =-\frac{12\mu AL^{2}}{\mu IJ_{m}},\hat{F}=\frac{FL^{3}}{\mu I}$$

Utilizing the above equations, the dimensionless partial differential equation governing the transverse vibration of the beams is obtained as (the hat notation is omitted for convenience).

(18) $$\frac{\partial^{2}w}{\partial t^{2}}+c\frac{\partial w}{\partial t}+\frac{1}{J_{m}}\frac{\partial^{4}w}{\partial x^{4}}+\eta_{1}\frac{\partial^{4}w}{\partial x^{4}}+\eta_{2}\frac{\partial^{2}w}{\partial x^{2}}+\beta {\left(\frac{\partial w}{\partial x}\right)}^{2}\frac{\partial^{2}w}{\partial x^{2}}=F\cos\left(\Omega t\right)$$

Subsequently, the boundary conditions become

(19) $$w\left(0\right)=0,\;w\left(1\right)=0,\;\frac{dw\left(0\right)}{dx}=0,\;\frac{dw\left(1\right)}{dx}=0$$

The system is continuous, and therefore there are infinite modes of vibration. In this paper, the first mode is considered only, with the aid of the separation of variable technique and the Galerkin method. Based on the separation of variable technique, we assume the transverse response is approximated as

(20) w(x,t)=W(x)q(t)

in which q(t) is the time-dependent coordinate of vibration; W(x) stands for the mode shape of a double-clamped beam that is given below [56]

(21) $$W\left(x\right)=\sqrt{\frac{2}{3}}\;\left[1-\cos\left(2\;\pi \;x\right)\right]$$

The function expressed in Equation (21) satisfies conditions in Equation (19).

According to the Galerkin method, Equation (20) is substituted in Equation (18), and the resulting equation is multiplied by Equation (21), and integration over [0 1] is taken, which results in

(22) $$\ddot{q}+c\dot{q}+\omega_{0}^{2}q+\alpha q^{3}=f\cos\left(\Omega t\right)$$

In which

(23) $$\omega_{0}={\left(\frac{\int_{0}^{1}\left\{\eta_{1}W^{⁗}W+\frac{1}{J_{m}}W^{⁗}W+\eta_{2}W^{\prime \prime }W\right\}dx}{\int_{0}^{1}W^{2}dx}\right)}^{\frac{1}{2}}\;\alpha =\frac{\int_{0}^{1}\left(\beta W^{\prime }W^{\prime }W^{\prime \prime }W\right)dx}{\int_{0}^{1}W^{2}dx}\;f=\frac{\int_{0}^{1}\left(FW\right)dx}{\int_{0}^{1}W^{2}dx}$$

In Equation (23), $\omega_{0}$ indicates dimensionless linear natural frequency.

## 3. Solution Method

This section is divided into two parts. In the first one, the forced vibration is solved using the Multiple Scales Method (MSM) [57], and in the second one, the free vibration is solved using Hamilton Approach (HA).

### 3.1. Forced Vibration Solution

To implement the MSM, the forced vibration equation, Equation (22), is converted to a perturbated form by introducing the following parameters

(24) $$c=2\varepsilon \;c_{d},\;\alpha =\varepsilon \alpha_{1},\;f=\varepsilon \;f_{1}$$

where ε is a dimensionless quantity that measures the strength of the nonlinearity of the beam and is called the gauge parameter.

Substituting Equation (24) into Equation (22), we obtain

(25) $$\ddot{q}+2\varepsilon \;c_{d}\dot{q}+\omega_{0}^{2}q+\varepsilon \alpha_{1}q^{3}=\varepsilon \;f_{1}\cos\left(\Omega t\right)$$

In line with the MSM, the original time is replaced with new time scales as $T_{n}=\varepsilon^{n}\;t;n=1,2,\ldots$ and therefore, the ODE is converted to a PDE.

New differential operators based on new time scales are $D_{n}=\partial /\partial T_{n}$, and original time first and second derivatives in terms of these operators are expressed as

(26) $$\frac{d}{dt}=D_{0}+\varepsilon \;D_{1}+\varepsilon^{2}\;D_{2}+\ldots \;\frac{d^{2}}{dt^{2}}=D_{0}{}^{2}+2\;\varepsilon \;D_{0}D_{1}+\varepsilon^{2}\left(D_{1}^{2}+2\;D_{0}D_{2}\;\right)+\ldots$$

The governing equation includes a nonlinear cubic term. Therefore, a first-order perturbation approximation is accurate enough, such that

(27) $$q=q_{0}+\varepsilon \;q_{1}$$

$q_{n},\;n=0,1$ are independent of the gauge parameter ε. For this reason, we can equal the coefficient of each power of ε to zero.

Combining Equations (25)–(27), and equating coefficients of $\varepsilon^{0}$ and $\varepsilon^{1}$ to zero, the following PDEs are attained

Coefficients of $\varepsilon^{0}$

(28) $$D_{0}^{2}q_{0}+\omega_{0}^{2}q_{0}=0$$

Coefficients of $\varepsilon^{1}$

(29) $$D_{0}^{2}q_{1}+\omega_{0}^{2}q_{1}=-2D_{0}D_{1}q_{0}-2D_{0}q_{0}-\alpha_{1}q_{0}{}^{3}+f_{1}\cos\left(\Omega t\right)$$

The solution of Equation (28) takes the following form

(30) $$q_{0}=A\left(T_{1}\right)e^{i\;\omega_{0}T_{0}}+\bar{A}\left(T_{1}\right)e^{-i\;\omega_{0}T_{0}}$$

in which $A\left(T_{1}\right)$ is a complex-valued function and $\bar{A}\left(T_{1}\right)$ is its complex conjugate.

Substituting Equation (30) into Equation (29), the following equation is obtained as

(31) $$D_{0}^{2}q_{1}+\omega_{0}^{2}q_{1}=\left[-3\alpha_{1}A^{2}\bar{A}-2ic_{d}A\omega_{0}-2i\omega_{0}\frac{dA}{dT_{1}}\right]e^{i\;\omega_{0}T_{0}}+f_{1}\cos\left(\Omega t\right)+\mathrm{CC}+\mathrm{NST}$$

In the above equation, the terms inside the box bracket shows secular terms, CC stands for complex conjugates of previous terms, and NST is an abbreviation for terms with higher degrees of $e^{i\;\omega_{0}T_{0}}$ (nonsecular terms).

By equating secular terms to zero, the frequency-amplitude relation can be obtained. However, the external loading can also give rise to secure terms. This fact is considered in two states, i.e., the primary resonance and the secondary resonance. In this paper, the primary resonance is analyzed, which states that

(32) $$\Omega =\omega_{0}+\varepsilon \;\sigma$$

Writing the trigonometric function in Equation (31) and using Equation (32), we obtain

(33) $$3\alpha_{1}A^{2}\bar{A}+2ic_{d}A\omega_{0}+2i\omega_{0}\frac{dA}{dT_{1}}-\frac{1}{2}f_{1}e^{i\sigma T_{1}}=0$$

The complex-valued function A is written as

(34) $$A=\frac{1}{2}ae^{i\theta },\;\bar{A}=\frac{1}{2}ae^{-i\theta }$$

in which a and θ are the amplitude and phase, which are functions of $T_{1}$.

Substituting Equation (34) into Equation (33) and then separating the resulting equation into real and imaginary parts yields

Imaginary parts:

(35) $$\frac{\mathrm{da}}{{\mathrm{dT}}_{1}}=-ac_{d}+\frac{1}{2\omega_{0}}f_{1}\sin\left(\sigma T_{1}-\theta \right)$$

Real parts:

(36) $$a\frac{d\theta }{dT_{1}}=\frac{3}{8\omega_{0}}\alpha_{1}a^{3}-\frac{1}{2\omega_{0}}f_{1}\cos\left(\sigma T_{1}-\theta \right)$$

Equations (35) and (36) are converted to an autonomous equation by introducing $\gamma =\left(\sigma T_{1}-\theta \right)$, which results in

(37) $$\frac{\mathrm{da}}{{\mathrm{dT}}_{1}}=-ac_{d}+\frac{1}{2\omega_{0}}f_{1}\sin\left(\gamma \right)$$

(38) $$a\frac{d\gamma }{dT_{1}}=\sigma a-\frac{3}{8\omega_{0}}\alpha_{1}a^{3}+\frac{1}{2\omega_{0}}f_{1}\cos\left(\gamma \right)$$

A bounded response is acquired while $\frac{\mathrm{da}}{{\mathrm{dT}}_{1}}=a\frac{d\gamma }{dT_{1}}=0$, whereby one can obtain

(39) $$ac_{d}=\frac{1}{2\omega_{0}}f_{1}\sin\left(\gamma \right)$$

(40) $$\sigma a-\frac{3}{8\omega_{0}}\alpha_{1}a^{3}=-\frac{1}{2\omega_{0}}f_{1}\cos\left(\gamma \right)$$

After some mathematical manipulation and using the fact $\sin^{2}\left(\gamma \right)+\cos^{2}\left(\gamma \right)=1$, we obtain the frequency-amplitude response as

(41) $${\left[c_{d}a\right]}^{2}+{\left[\sigma a-\frac{3}{8\omega_{0}}\alpha_{1}a^{3}\right]}^{2}={\left[\frac{1}{2\omega_{0}}f_{1}\right]}^{2}$$

### 3.2. Free Vibration Solution

In this subsection, the nonlinear frequency of the micro/nanobeam with neglecting the external force and damping is obtained via Hamilton’s approach. The initial conditions for the vibration of the hyperelastic beam are expressed as

(42) $$q\left(0\right)=a_{0},\;\dot{q}\left(0\right)=0$$

where $a_{0}$ stands for the maximum amplitude of the vibration. Based on Hamilton’s principle, the nonlinear frequency is derived as [58]

(43) $$\omega_{nl}=\sqrt{\omega_{0}^{2}+\frac{49}{70}\alpha a_{0}^{2}}$$

## 4. Result and Discussion

The effects of several parameters such as the stiffening parameter, the length scale parameter, and forcing amplitude and damping on the frequency response and nonlinear frequency of the system are analyzed. The material and geometrical parameters of the hyperelastic microbeam are given in Table 1.

### 4.1. Frequency Response

**Figure 2:**
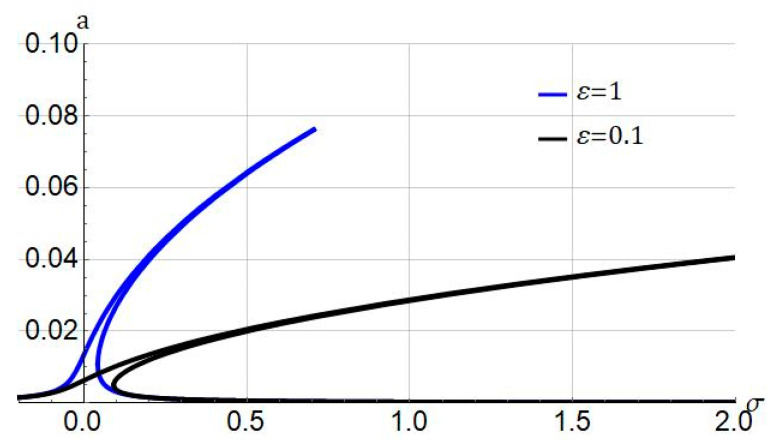
Influence of gauge parameter (ε) on frequency response of system. Systems parameters: ℓ=0; $c_{d}=0.004$; $J_{m}=100$; $f_{1}=0.5$.

Figure 2 depicts the influence of the gauge parameter ε on the frequency response under the following parameter $f_{1}=0.5$, ℓ=0, $c_{d}=0.004$, and $J_{m}=100$. As the gauge parameter ε is decreased, the nonlinearity of the system increases. Mathematically speaking, with the decrease of ε, the value of nonlinear terms in the equation of motion becomes higher in comparison to the value of linear terms. Depending on the accuracy, an arbitrary value for ε can be adopted, which in this paper it is chosen as ε=1.

**Figure 3:**
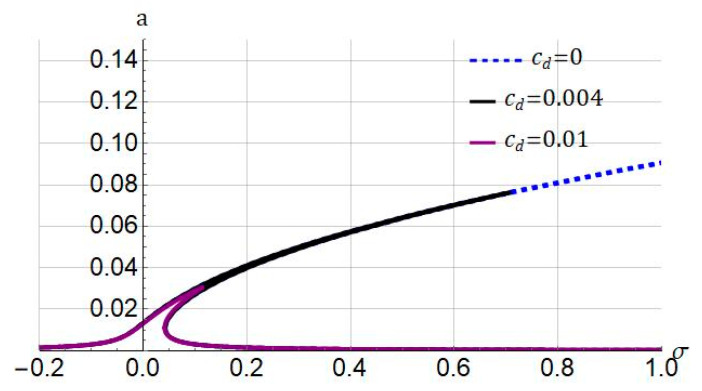
Influence of damping coefficient ($c_{d}$) on frequency response of system. Systems parameters: ℓ=0; $J_{m}=100$; $f_{1}=0.5$.

Illustrated in Figure 3 is the influence of the damping coefficient $c_{d}$ on the frequency response of the system while considering the following parameters ℓ=0, and $J_{m}=100$. From the figure, it is concluded that increasing the damping coefficient decreases the response amplitude of the hyperelastic micro/nanobeam. The damping in hyperelastic materials mainly originates from the viscosity of matter. In the remaining part of the numerical simulation, as a test case, the damping coefficient is taken as $c_{d}=0.004$.

**Figure 4:**
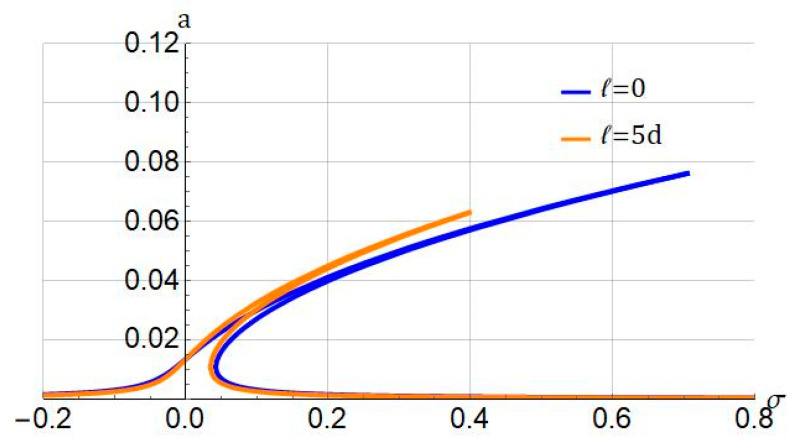
Influence of length scale parameter (ℓ) on frequency response of system. Systems parameters: $J_{m}=100$; $f_{1}=0.5$; $c_{d}=0.004$.

Figure 4 represents the impact of the length scale parameter ℓ on the nonlinear resonant vibration of the hyperelastic beam. As seen, increasing the size effect, the response amplitude decreases, and the hardening nonlinearity becomes weaker. This result is in agreement with that shown in the literature for linear materials. Obtaining the accurate small-length scall parameter in the experimental test is a crucial task for engineers. Finding an exact value for the length scale parameter for the hyperelastic beam in the experimental test should be carried out to improve the performance of hyperelastic microbeams.

**Figure 5:**
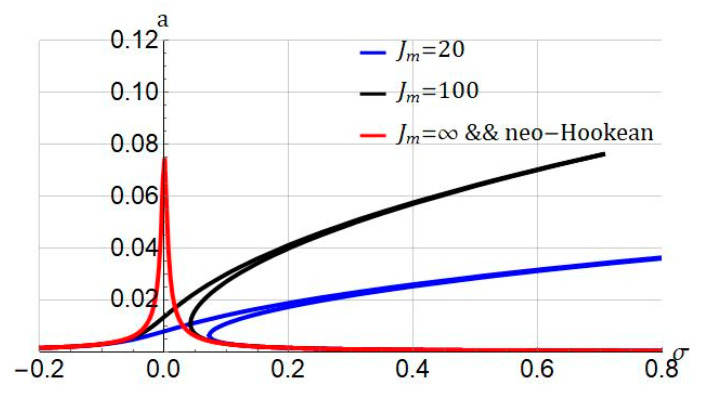
Influence of stiffening parameter ($J_{m}$) on frequency response of system. Systems parameters: ℓ=0; $f_{1}=0.5$; $c_{d}=0.004$.

The influence of the stiffening parameter $J_{m}$ on the frequency-amplitude plot is shown in Figure 5. It is concluded that as the stiffening parameter is decreased, the hardening nonlinearity gets stronger. When the stiffening parameter is equal to $J_{m}=\infty$, i.e., the conversion of the Gent model to the neo-Hookean model, the system’s response is linear. It is noted that if the stiffening parameter is smaller, the strain-stiffening effect is stronger. As reported by Amabili, a stiffening parameter in a range $J_{m}=30-100$ stands for rubber materials, and values less than them stand for biological tissues [12].

**Figure 6:**
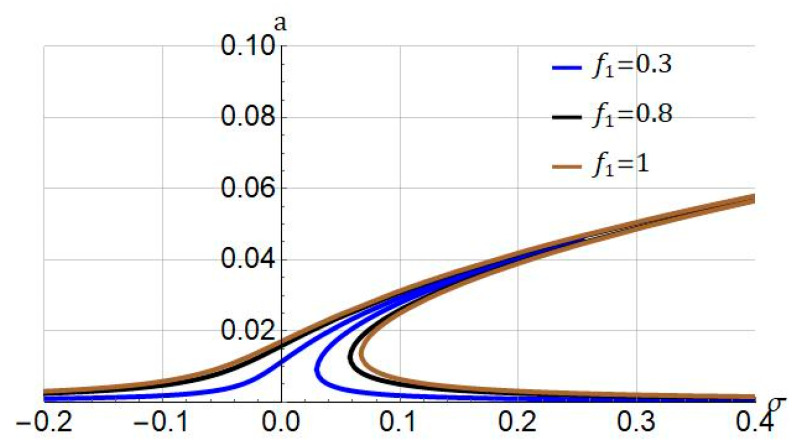
Influence of forcing amplitude ($f_{1}$) on frequency response of system. Systems parameters: ℓ=0; $J_{m}=100$; $c_{d}=0.004$.

The influence of the amplitude of the external loading $f_{1}$ on the resonant characteristics of the hyperelastic micro/nanobeam is analyzed in Figure 6. Increasing $f_{1}$ the response amplitude increases, and the frequency response becomes wider. Moreover, the forcing amplitude cannot alter the nonlinear nature of the system and only quantitatively alter the resonant behaviour.

**Figure 7:**
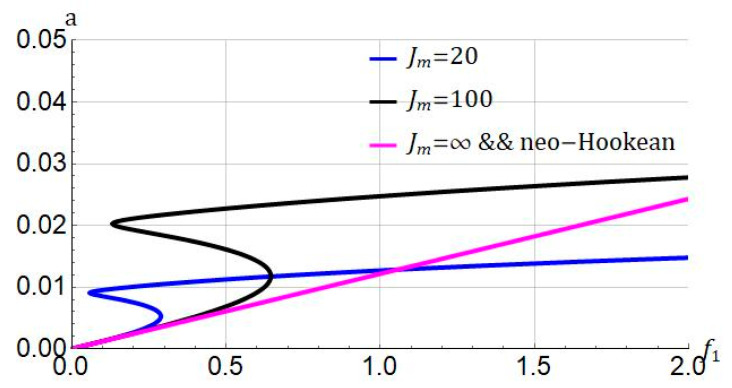
Influence of stiffening parameter ($J_{m}$) on force response of system. Systems parameters: ℓ=0; σ=0.05; $c_{d}=0.004$.

We analyze the influence of the strain-stiffening parameter on the force-response in Figure 7. The system parameters are ℓ=0; σ=0.05; $c_{d}=0.004$. We can see that by increasing the value of the strain-stiffening parameter, a higher value of forcing amplitude is required to cause the jump phenomenon. Moreover, by increasing the strain-stiffening parameter, the system becomes stable and for the neo-Hookean model.

**Figure 8:**
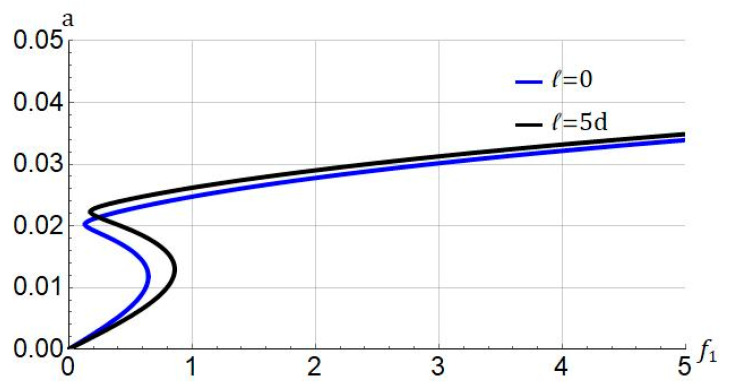
Influence of stiffening parameter (ℓ) on force response of system. Systems parameters: $J_{m}=100$; σ=0.05; $c_{d}=0.004$.

We show the influence of the length-scale parameter on the force-response in Figure 8. With the inclusion of the effect of size, the jump phenomenon arises for higher values of forcing amplitude.

### 4.2. Nonlinear Frequency

The previous section demonstrates the results for the forced vibration of the Gent hyperelastic beam. Herein, the nonlinear frequency of the system given in Equation (42) is evaluated.

**Figure 9:**
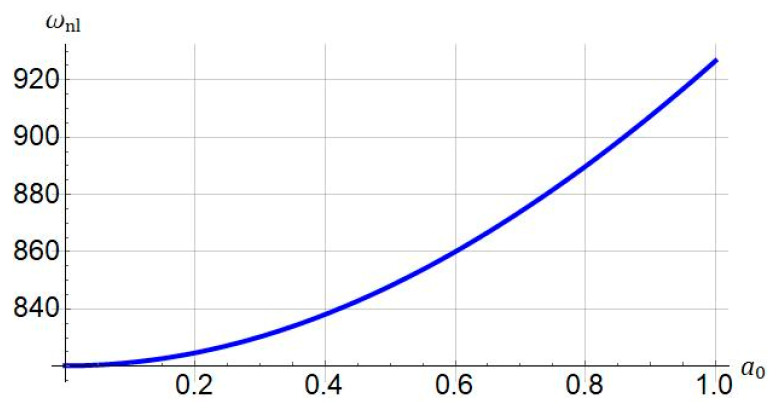
Influence of maximum amplitude ($a_{0}$) on nonlinear frequency of system. Systems parameters: ℓ=0; $J_{m}=100$, L=30 μm.

Illustrated in Figure 9 is the nonlinear frequency versus the maximum amplitude when ℓ=0, and $J_{m}=100$. It is found that by increasing the maximum amplitude $a_{0}$ the nonlinear frequency increases.

**Figure 10:**
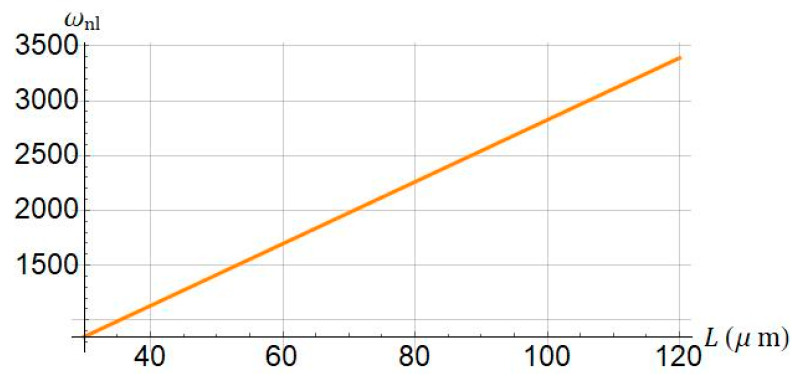
Influence of length of micro/nanobeam (L) on nonlinear frequency of system. Systems parameters: ℓ=0; $J_{m}=100$; $a_{0}=0.5$.

As depicted in Figure 10, the nonlinear frequency for variations of the length of the beam is presented. As the length is increased, the dimensionless nonlinear frequency increases accordingly.

**Figure 11:**
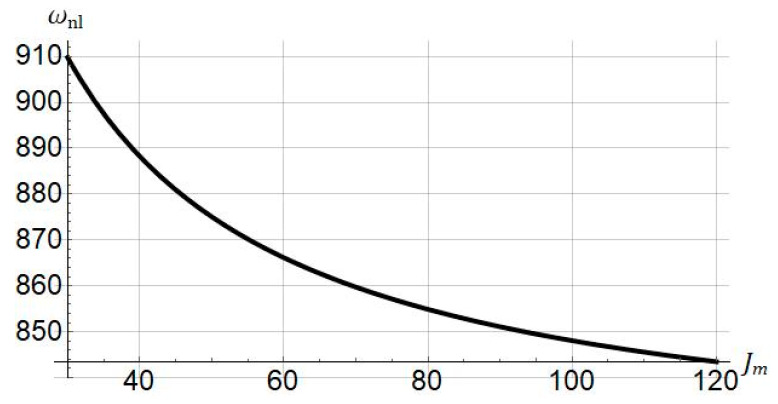
Influence of stiffening parameter ($J_{m}$) on nonlinear frequency of system. Systems parameters: L=30 μm; ℓ=0; $a_{0}=0.5$.

The nonlinear frequency versus the stiffening parameter $J_{m}$ is presented in Figure 11. Increasing $J_{m}$, the nonlinear frequency decreases.

**Figure 12:**
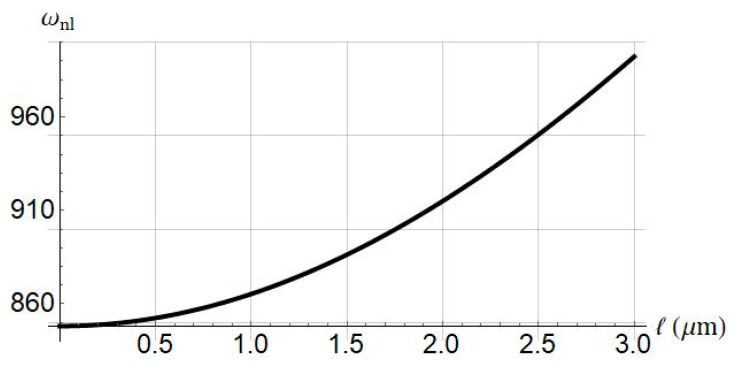
Influence of length scale parameter ℓ on nonlinear frequency of system. Systems parameters: L=30 μm; $J_{m}=100$; $a_{0}=0.5$.

As depicted in Figure 12, the nonlinear frequency versus the length scale parameter is presented. As the size effect is increased, the nonlinear frequency increases accordingly.

## 5. Discussion on the Strain-Stiffening

Rubber-like materials can be deformed by stretching. In the beginning, we can stretch rubbers easily, but if the stretch is large enough, the stretching process becomes difficult. This is due to the strain-stiffening effect in rubber-like materials. The strain-stiffening is a nonlinear behavior that is seen even in soft biological materials such as liver and brain tissue [59]. We can use this property in hyperelastic materials so as to evade damage. The strain-stiffening can also be connected to the molecular-statistical point of view in nonlinear elasticity. The stiffening parameters $J_{m}$ in the Gent model relates to the number of rigid links in a single chain N using $J_{m}=3\left(N-1\right)$. N is also called the classical number of Kuhn segments [60]. The results of Figure 5, Figure 7 and Figure 11, can also be interpreted based on molecular-statistical point of view. We see that altering $J_{m}$, the number of segments changes accordingly. Therefore, this change affects the frequency/force response of the hyperelastic microbeam. Taken together, the results of this paper can help researchers who would like to analyze the hyperelastic microbeam via molecular-statistical hyperelastic models such as generalized neo-Hookean model.

## 6. Conclusions

In this paper, nonlinear, free, and forced oscillations of a hyperelastic micro/nanobeam were investigated with the inclusion of the small-scale effect, strain-stiffening effect, and moderate rotation. A developed Euler–Bernoulli beam theory was utilized to model the beam, and the energies and works that appeared in the system were formulated. The equation of motion was derived using Hamilton’s principle and the Galerkin decomposition method. Frequency-amplitude curves and the nonlinear natural frequency diagrams were illustrated by analytically solving the equation of motion. This paper concludes that:

- Increasing the strain-stiffening effect leads to increasing hardening nonlinearity.
- For the neo-Hookean model with $J_{m}=\infty$, the nonlinearity vanishes, and the response is transformed into a linear type.
- As the stiffening parameter $J_{m}$ is increased, the nonlinear natural frequency decreases.
- The length of the micro/nanobeam, the damping, and size effects were identified as influential parameters in the system.

## Figures and Tables

**Table 1:** Material and geometrical parameters.

Parameters	Value
Length	L=30 μm
Width	b=10 μm
Height	d=0.65 μm
Young’s modulus	E=3 GPa
Shear modulus	μ=E/3=1 GPa

## Data Availability

Data sharing is not applicable.
